# Histone acetyltransferase Sas3 contributes to fungal development, cell wall integrity, and virulence in *Aspergillus fumigatus*

**DOI:** 10.1128/aem.01885-23

**Published:** 2024-03-07

**Authors:** Yamei Wang, Jialu Fan, Zhengyu Zhou, Gustavo H. Goldman, Ling Lu, Yuanwei Zhang

**Affiliations:** 1 Jiangsu Key Laboratory for Microbes and Functional Genomics, Jiangsu Engineering and Technology Research Centre for Microbiology, College of Life Sciences, Nanjing Normal University, Nanjing, China; 2 Faculdade de Ciências Farmacêuticas de Ribeirão Preto, Universidade de São Paulo, Ribeirão Preto, Brazil; Chalmers tekniska hogskola AB, Gothenburg, Sweden

**Keywords:** *Aspergillus fumigatus*, histone acetyltransferase, Sas3, MYST family, cell wall integrity, virulence

## Abstract

Histone acetyltransferase (HAT)-mediated epigenetic modification is essential for diverse cellular processes in eukaryotes. However, the functions of HATs in the human pathogen *Aspergillus fumigatus* remain poorly understood. In this study, we characterized the functions of MOZ, Ybf2/Sas3, Sas2, and Tip60 (MYST)-family histone acetyltransferase something about silencing (Sas3) in *A. fumigatus*. Phenotypic analysis revealed that loss of Sas3 results in significant impairments in colony growth, conidiation, and virulence in the *Galleria mellonella* model. Subcellular localization and Western blot analysis demonstrated that Sas3 localizes to nuclei and is capable of acetylating lysine 9 and 14 of histone H3 *in vivo*. Importantly, we found that Sas3 is critical for the cell wall integrity (CWI) pathway in *A. fumigatus* as evidenced by hypersensitivity to cell wall-perturbing agents, altered cell wall thickness, and abnormal phosphorylation levels of CWI protein kinase MpkA. Furthermore, site-directed mutagenesis studies revealed that the conserved glycine residues G641 and G643 and glutamate residue E664 are crucial for the acetylation activity of Sas3. Unexpectedly, only triple mutations of Sas3 (G641A/G643A/E664A) displayed defective phenotypes similar to the Δ*sas3* mutant, while double or single mutations did not. This result implies that the role of Sas3 may extend beyond histone acetylation. Collectively, our findings demonstrate that MYST-family HAT Sas3 plays an important role in the fungal development, virulence, and cell wall integrity in *A. fumigatus*.

**IMPORTANCE:**

Epigenetic modification governed by HATs is indispensable for various cellular processes in eukaryotes. Nonetheless, the precise functions of HATs in the human pathogen *Aspergillus fumigatus* remain elusive. In this study, we unveil the roles of MYST-family HAT Sas3 in colony growth, conidiation, virulence, and cell wall stress response in *A. fumigatus*. Particularly, our findings demonstrate that Sas3 can function through mechanisms unrelated to histone acetylation, as evidenced by site-directed mutagenesis experiments. Overall, this study broadens our understanding of the regulatory mechanism of HATs in fungal pathogens.

## INTRODUCTION

Post-translational histone modifications, such as acetylation, methylation, phosphorylation, sumoylation, and ubiquitination, have critical roles in multiple fundamental cellular processes through regulation of the chromatin structure and gene expression (1–3). Histone acetylation is a reversible dynamic process that is mediated by a fine-tuned balance between histone acetyltransferases (HATs) and histone deacetylases (4). HATs add an acetyl group to lysine residues of histone tails and neutralize their positive charge, thus increasing the accessibility of nucleosomal DNA to transcription factors and coregulatory proteins (5). The HAT proteins are grouped into three major families, including the Gcn5-related N-acetyltransferase family, the p300/CBP family (p300 and CREB-binding protein), and the MOZ, Ybf2/Sas3, Sas2, and Tip60 (MYST) family based on structural and sequence homology (6, 7). Overwhelming evidence has shown that HATs participate in the regulation of several aspects of physiological processes in eukaryotes (8). Therefore, exploring the role and regulatory mechanism of HATs will deepen the understanding of post-translational modifications.

The filamentous fungus *Aspergillus fumigatus* is a notorious opportunistic fungal pathogen that causes several respiratory diseases, such as allergic bronchopulmonary aspergillosis and invasive pulmonary aspergillosis, with high mortality rates among immunocompromised patients (9). Although *A. fumigatus* is a soil-borne saprophytic fungus, its biological characteristics from the physiological adaptation to harsh environments, such as thermotolerance, confer virulence to mammalian hosts (10, 11). Chromatin structure and gene expression that are mediated by histone acetylation play an important role in the virulence potential of fungal pathogens. Several HATs have been proven essential for the growth, development, virulence, and host adaptation of fungal pathogens. For example, *Fusarium graminearum* histone acetyltransferase Gcn5 cooperates with the transcription factor FgPacC30 in an iron-rich environment to protect cells from high iron toxicity (12). In *Cryptococcus neoformans*, Gcn5-mediated histone H3 acetylation plays a pivotal role in completing the sexual cycle, yeast-hyphae morphogenesis, and sexual reproduction (13). Orthologs of fungal-specific HAT Rtt109 regulate fungal development, stress tolerance, and virulence in *A. fumigatus* (14); morphogenesis and aflatoxin synthesis in *Aspergillus flavus* (15); and antifungal drug sensitivity in *Candida albicans* (16).

Something about silencing (Sas3) belongs to the MYST family of HATs and is a component of the nucleosomal acetyltransferase of histone H3 (NuA3) complex (17, 18). As the catalytic subunit of the NuA3 complex, Sas3 plays an essential role in HAT activity and in maintaining the integrity of the NuA3 complex. In *Saccharomyces cerevisiae*, Sas3 is required for the acetylation of H3K9 and H3K14 *in vivo*. However, disrupting *sas3* does not yield any notable phenotypic alterations (19). In contrast to the phenotype of *sas3* null mutant in yeast, loss of Sas3 orthologs in plant and insect pathogens *Magnaporthe oryzae*, *Fusarium graminearum*, *Zymoseptoria tritici*, and *Beauveria bassiana* leads to impaired hyphal growth, decreased asexual sporulation, and reduced pathogenicity (20–23). Deleting the Sas3 ortholog Hat1 in *Metarhizium robertsii* results in a reduction in H3 acetylation and the subsequent activation of secondary metabolite genes (24). In *Aspergillus flavus*, MystB, an ortholog of Sas3, mediates secondary metabolite and virulence by its histone acetylation activity (25). Together, histone acetyltransferase Sas3 regulates a diverse array of biological processes across multiple pathogenic fungi. However, the biological function of Sas3 and its underlying molecular mechanisms in *A. fumigatus* remain unclear and need to be elucidated.

In the present study, we characterized the biological functions of Sas3 in *A. fumigatus*. We showed that nucleus-localized Sas3 possesses histone acetyltransferase activity that catalyzes acetylation of histone H3K9 and K14 *in vivo*. Sas3 is responsible for the regulation of hyphal growth, conidiation, virulence, and cell wall stress response in *A. fumigatus*. In line with phenotypic defects of *sas3* null mutants, the transcriptomic data revealed that Sas3 regulates the expression of genes involved in carbohydrate metabolism, amino acid biosynthesis, cell wall integrity pathway, and conidiation. Furthermore, our findings demonstrate that the conserved catalytic sites in the HAT domain (G641, G643, and E664) are necessary for Sas3 acetylation activity, but only simultaneous mutation of all three conserved residues resulted in a phenotype resembling that of the Δ*sas3* mutant, implying that Sas3 may possess additional functional roles beyond histone acetylation in *A. fumigatus*. Our findings will improve our understanding of the underlying regulatory mechanism of HATs in fungal pathogens.

## RESULTS

### Sas3 affects vegetative growth, conidiation, and virulence in *A. fumigatus*

**Fig 1:**
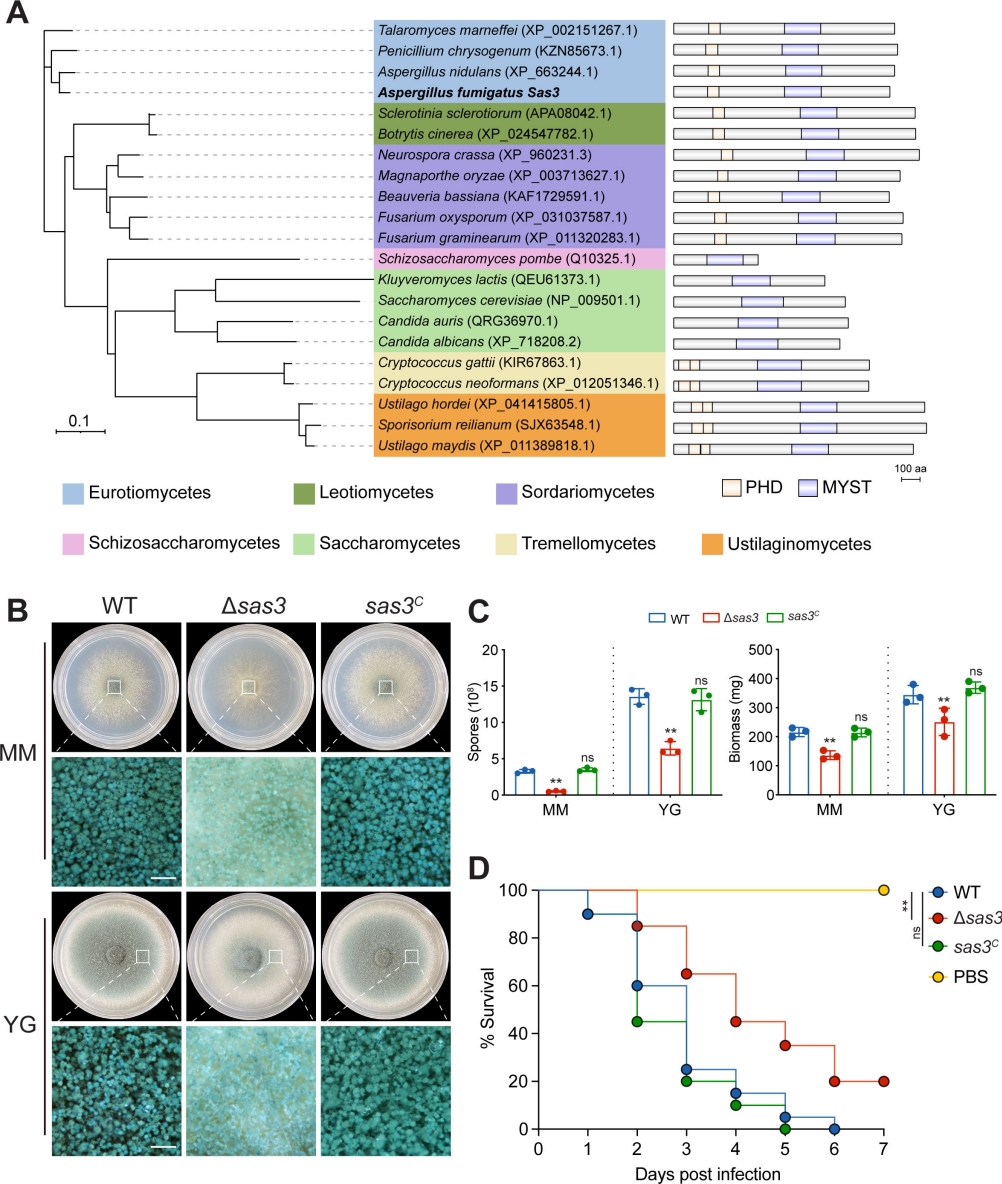
*A. fumigatus* Sas3 is required for vegetative growth, conidiation, and virulence. (**A**) Neighbor-joining phylogenetic tree and schematic diagram of the domain architecture of Sas3 orthologs in different fungal species. Scale bar (0.1) indicates genetic distance. (**B**) Representative growth phenotypes of the wild-type, Δ*sas3*, and complementation strains on solid MM and complete YG medium at 37°C for 48 h. Stereomicroscope imaging shows the close-up views of conidiation patterns from the selected area of the indicated strains (white box). Scale bar = 10 µm. (**C**) Quantitative examination of conidia production and biomass of the indicated strains. Data from three independent experiments are presented as mean ± SD. Statistical analysis was performed using two-tailed, unpaired *t*-tests. (**D**) Survival curves for *G. mellonella* larvae infected with the wild-type, Δ*sas3*, and complementation strains. Each experiment was replicated three times independently. Statistical analysis was performed using the log-rank test. **P < 0.01. MM, minimal medium; ns, not significant; PBS, phosphate-buffered saline.

A BLASTp search utilizing *S. cerevisiae* Sas3 against the *A. fumigatus* A1163 genome database was used to identify the probable Sas3 ortholog. As a result, we found only one candidate for the putative histone acetyltransferase Sas3 (AFUB 067970, National Center for Biotechnology Information accession number EDP50460.1) in *A. fumigatus*. The *sas3* open reading frame in *A. fumigatus* is 3,336 bp long, with two introns, and is expected to encode a 1,058-amino acid protein. The N-terminal region of *A. fumigatus* Sas3 contains a plant homeodomain (PHD) zinc finger and a MYST domain, according to domain architectural analysis (Fig. 1A). Sas3 is highly conserved in divergent fungus species, and these two domains are conserved in all fungal Sas3-like proteins, with the exception of *Saccharomycetes* and *Schizosaccharomycetes*, which lack the PHD zinc finger domain (Fig. 1A). To investigate biological functions of Sas3, we generated the Δ*sas3* null mutant and complemented strains. The mutants were verified by diagnostic PCR analysis (Fig. S1). On the solid minimal medium (MM) and rich medium YG (yeast extract with glucose), the Δ*sas3* mutant displayed drastically reduced conidiation and hyphal development compared to wild type (WT) and complemented strains (Fig. 1B). Quantitative analyses of conidia formation on solid medium revealed that the number of conidia of the Δ*sas3* mutant was dramatically decreased to approximately 16% and 47% of that in WT and complemented strains on MM and YG medium, respectively (Fig. 1C). Moreover, the Δ*sas3* mutant displayed a considerably lower biomass in submerged liquid MM and YG media (Fig. 1C), which corresponded to the reduced hyphal development in solid medium. To investigate whether Sas3 influences the virulence of *A. fumigatus*, we used the *Galleria mellonella* wax moth infection model to assess the virulence potential of the wild-type strain, Δ*sas3*, and complementary strains. As shown in Fig. 1D, the Δ*sas3* exhibited a significantly reduced mortality rate of larvae compared to the wild-type strain, whereas the complementary strain showed similar mortality rates as the wild-type strain. Together, these results suggested that Sas3 is necessary for hyphal growth, conidiation, and virulence in *A. fumigatus*.

### Nucleus-localized Sas3 is histone acetyltransferase in *A. fumigatus*

**Fig 2:**
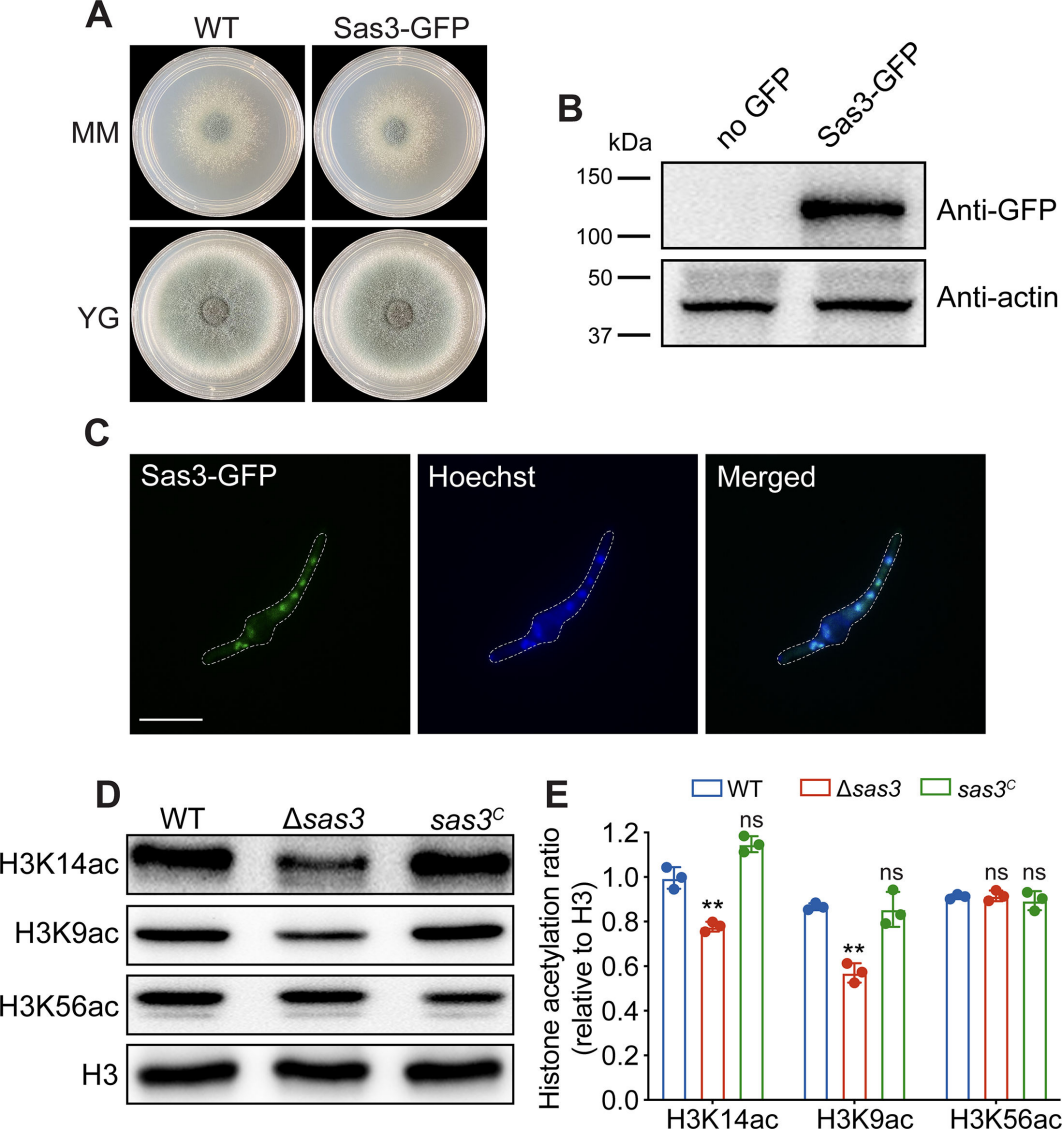
Nucleus-localized Sas3 possesses histone acetyltransferase activity. (**A**) Colony morphologies of the wild-type and Sas3-GFP strains grown on MM and YG media at 37°C for 48 h. (**B**) Western blot analysis of Sas3-GFP strain. β-Actin served as a loading control. (**C**) Localization of Sas3-GFP in vegetative stages of fungal development. Nuclei were stained with Hoechst dye 33258. Scale bar = 10 µm. (**D**) Western blotting for acetylated H3K9, H3K14, and H3K56 in the wild-type and Δ*sas3* mutants. H3 served as a loading control. (**E**) Quantified analysis of Western blot signal for H3K14ac, H3K9ac, and H3K56ac relative to H3 in the indicated strains. Data from three independent experiments are presented as mean ± SD. Statistical analysis was performed using two-tailed, unpaired *t*-tests. **P < 0.01. H3, histone H3; MAPK, mitogen-activated protein kinase; ns, not significant.

To examine the localization of Sas3 in *A. fumigatus*, we constructed the Sas3-green fluorescent protein (GFP) strain that labeled the C-terminus of Sas3 with a GFP under the control of its native promoter. When cultivated on MMs and rich media (YG), phenotypic examination revealed no differences between the Sas3-GFP strain and the parental wild-type strain, demonstrating that the C-terminal Sas3-GFP fusion protein is fully functional (Fig. 2A). Western blotting of the Sas3-GFP strain revealed a band of approximately 140 kDa, which corresponded to the mass of a fusion protein of GFP (27 kDa) and Sas3 (115 kDa), but no band was observed in the original wild-type strain (Fig. 2B). Sas3-GFP demonstrated robust nuclear localization in fluorescence microscopy, with green fluorescence from Sas3-GFP overlapping with blue fluorescence from nuclear stain Hoechst dye 33258 (Fig. 2C), suggesting that Sas3 consistently localizes in the nucleus in *A. fumigatus*. To investigate whether *A. fumigatus* Sas3 exhibits HAT activity, we determined histone acetylation levels by Western blot analysis using specific antibodies against acetylated H3-lysine 9, 14, and 56 (H3K9ac, H3K14ac, and H3K56ac), and an H3-specific antibody was used as a loading control. As shown in Fig. 2D and E, deletion of *sas3* resulted in decreased acetylation of histone H3K14 and H3K9, while no difference was observed in H3K56ac, which is in line with the findings observed in other Sas3 ortholog deletion mutants. Together, these results suggested that Sas3 localized in the nucleus and harbors histone acetyltransferase activity in *A. fumigatus*.

### Lack of Sas3 downregulates the expression of genes involved in cell wall integrity and conidiation

**Fig 3:**
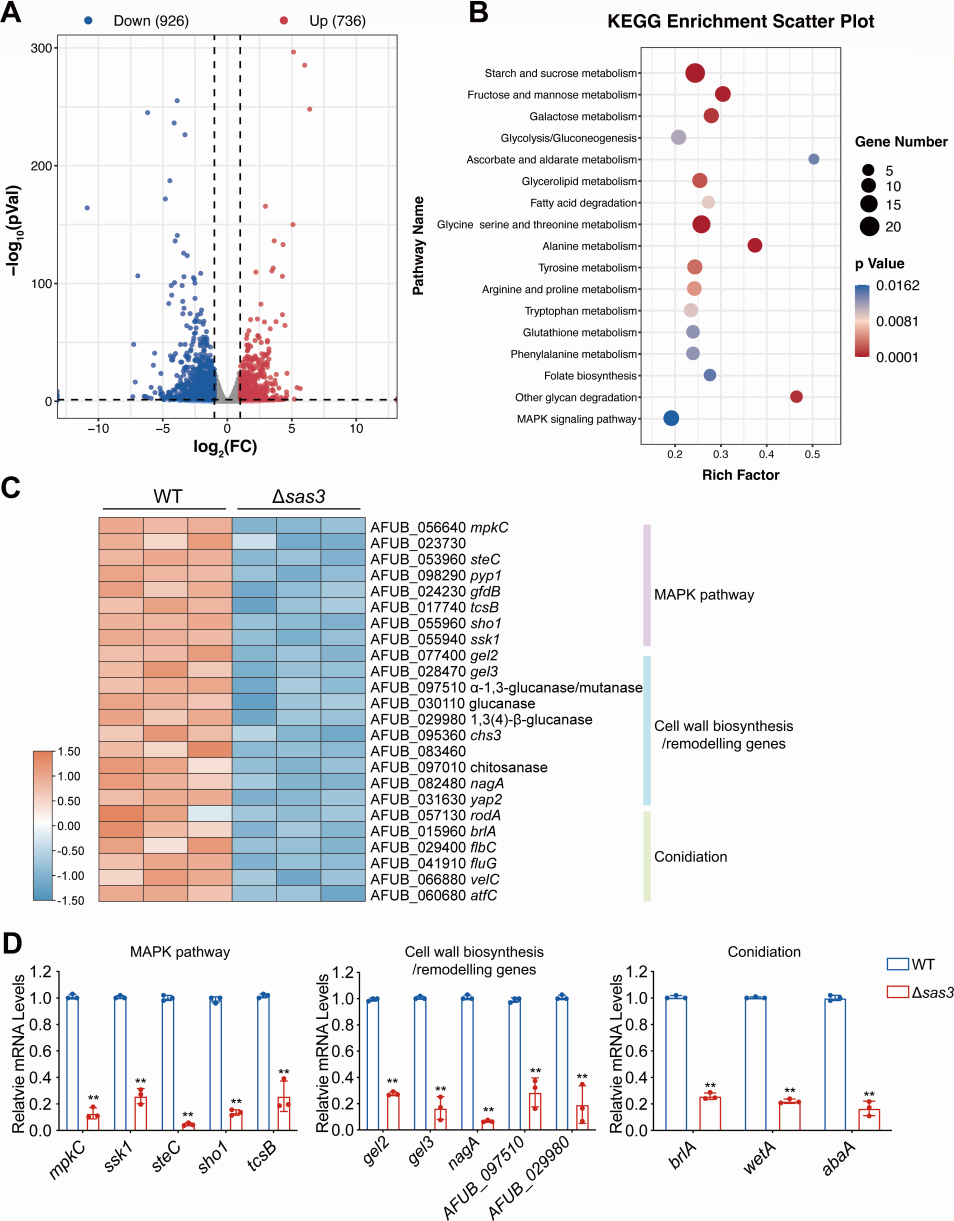
Loss of Sas3 leads to alterations in the expression of genes associated with cell wall integrity and conidiation. (**A**) The volcano plot showing the differentially expressed genes between the wild-type and Δ*sas3* mutants. (**B**) KEGG pathway enrichment analysis of differentially expressed genes between the wild-type and Δ*sas3* mutants. (**C**) A heatmap of the expression of cell wall biosynthesis- and conidiation-related genes between the wild-type and Δ*sas3* mutants. (**D**) The quantitative real-time analysis of selected genes in the wild-type and Δ*sas3* mutants. The mRNA levels were normalized to that of the reference gene *tubA*. Data from three independent experiments are presented as mean ± SD. Statistical analysis was performed using two-tailed, unpaired *t*-tests. **P < 0.01. KEGG, Kyoto Encyclopedia of Genes and Genomes.

To explore the mechanism by which Sas3 mediates hyphal growth and conidiation, we conducted RNA-seq analysis of wild-type and the Δ*sas3* strains grown in liquid MM. As shown in Fig. 3A, a total of 1,662 genes were significantly differentially expressed (log_2_ fold change ≥1.0 and ≤−1.0, *P* value < 0.05) in the Δ*sas3* mutant when compared to the wild-type strain, of which 736 were upregulated and 926 were downregulated (Table S3). Kyoto Encyclopedia of Genes and Genomes (KEGG) pathway enrichment analysis of all differentially expressed genes in the Δ*sas3* mutant showed the most significant terms of the pathway included carbohydrate metabolism, amino acid biosynthesis, and mitogen-activated protein kinase (MAPK) signaling pathway (Fig. 3B). Of particular interest were the significantly downregulated genes in the Δ*sas3* strain, including the genes required for cell wall integrity and remodeling (MAPK pathway components *mpkC*, *ssk1*, and *steC*, and cell wall biosynthesis genes *gel2*, *gel3*, and *nagA*) and conidiation (the key asexual developmental regulator *brlA*) (Fig. 3C). These results implied that these altered expression profiles may link to the phenotypic defects of the Δ*sas3* mutant. To verify the RNA-seq results, the selected genes were analyzed by quantitative real-time PCR (qRT-PCR). As shown in Fig. 3D, the qRT-PCR results revealed that the expression levels of selected genes were largely consistent with the RNA-seq data. Collectively, these results suggested that Sas3 plays an important role in the regulation of cell wall biosynthesis and conidiation.

### Sas3 is required for the maintenance of cell wall integrity in *A. fumigatus*

**Fig 4:**
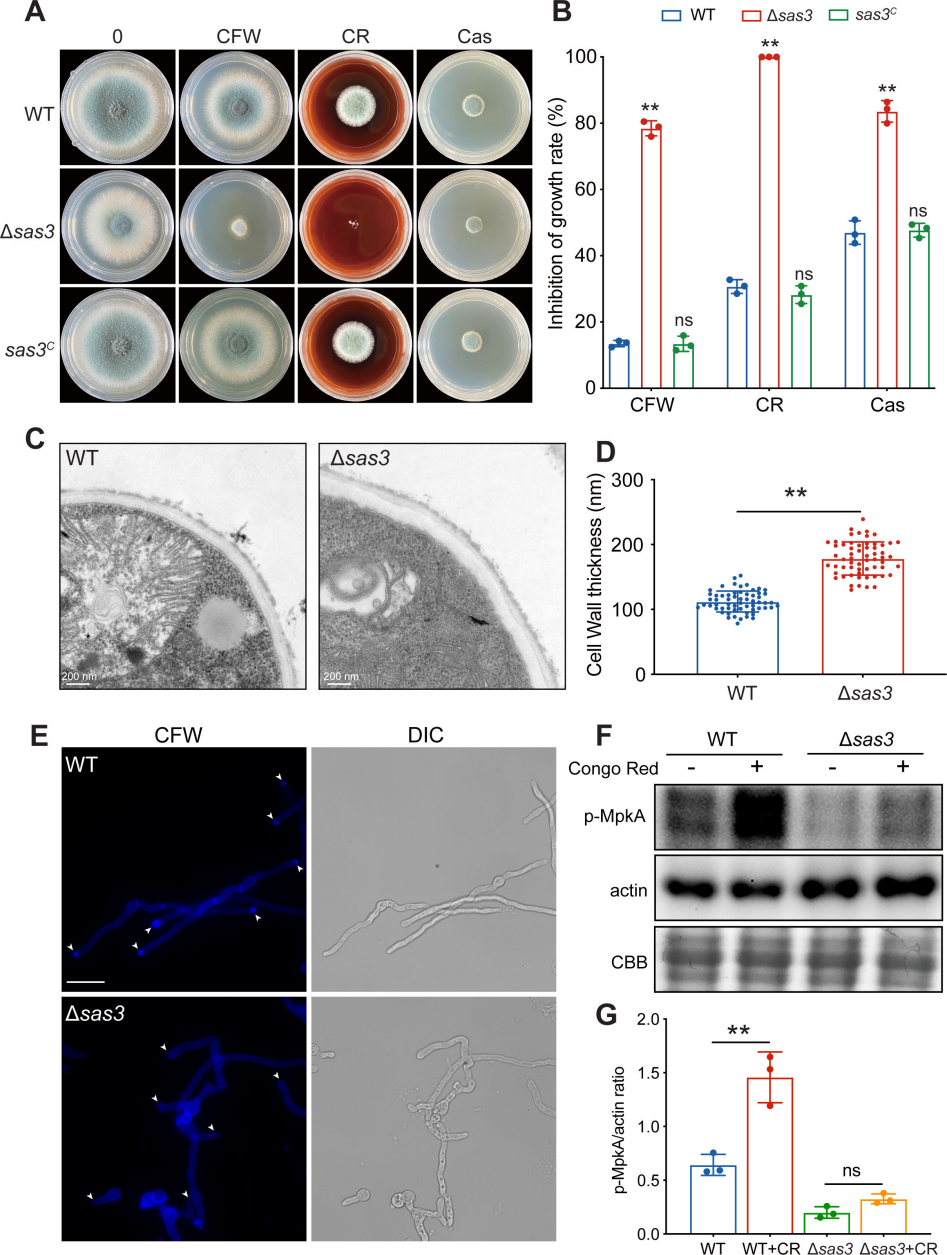
Sas3 is required for cell wall integrity in *A. fumigatus*. (**A**) Colony morphology of the wild-type, Δ*sas3*, and complementation strains grown on solid YG medium in the presence of 100-µg/mL calcofluor white, 200-µg/mL Congo red, and 0.5 µg/mL caspofungin at 37°C for 48 h. (**B**) Relative hyphal growth inhibition of the indicated strains grown on solid YG medium at 37°C for 48 h. Data are shown as mean ± SD from three independent experiments. Statistical analysis was performed using two-tailed, unpaired *t*-tests. (**C**) Representative transmission electron microscopy images of the wild-type and Δ*sas3* mutants. Scale bar = 200 nm. (**D**) Quantification of cell wall thickness of the wild-type and Δ*sas3* mutants. Data are shown as mean ± SD from three independent experiments. Statistical analysis was performed using two-tailed, unpaired *t*-tests. (**E**) The distribution of the chitin content in the wild-type and Δ*sas3* mutants was visualized through fluorescence microscopy with CFW staining. Hyphae of wild-type and Δ*sas3* mutants were stained with 5-mg/mL CFW for 1 min and then examined under a fluorescent microscope. The white arrowheads indicate the apex region of hyphal tips. Scale bar = 20 µm. (**F**) Western blot analysis of p-MpkAs in the wild-type and Δ*sas3* mutants in the presence and absence of CR. CBB staining and actin were used as the loading control. (**G**) Quantification of signal intensity ratio for p-MpkA to actin. Data are shown as mean ± SD from three independent experiments. Statistical analysis was performed using two-tailed, unpaired *t*-tests. **P < 0.01, ns, not significant; CBB, Coomassie brilliant blue; DIC, differential interference contrast; p-MpkA, phosphorylation level of MpkA.

To further evaluate the role of Sas3 for the cell wall integrity of *A. fumigatus*, we examined the sensitivity of the Δ*sas3* mutant to the cell wall-perturbing agents Congo red (CR, binds to glucan), calcofluor white (CFW, binds to chitin) (26), and caspofungin (Cas; β-1,3-glucan inhibitor) (27). As shown in Fig. 4A and B, Δ*sas3* mutant exhibited increased susceptibility to these agents compared to the wild type, while the complementary strain displayed similar phenotypes to the wild type under these stresses, suggesting that deletion of *sas3* results in cell wall defects in *A. fumigatus*. We further examined the cell wall structure by transmission electron microscopy, and the results showed that the cell wall of the Δ*sas3* mutant was thicker than the wild type (Fig. 4C and D). Additionally, fluorescence microscopy was conducted using CFW staining to determine chitin deposition in hyphae. CFW staining revealed a significantly lower intensity of CFW fluorescence in the apical region of hyphal tips in the Δ*sas3* mutant compared to that of the wild-type strain (Fig. 4E), suggesting that Sas3 is involved in the distribution of chitin in the cell wall. Due to the increased sensitivity to cell wall-perturbing compounds and altered cell wall thickness in the Δ*sas3* mutant, we further investigated the role of Sas3 in cell wall maintenance. The MAP kinase MpkA is the central regulator of CWI pathway in *A. fumigatus* and is phosphorylated under cell wall stresses (28, 29). To investigate whether Sas3 affects CWI pathway activation through MpkA phosphorylation, we evaluated the phosphorylation state of MpkA by Western blot using an anti-p44/42 MpkA antibody. As shown in Fig. 4F and G, the Δ*sas3* mutant displayed a significantly reduced phosphorylation level of MpkA compared to that of the wild type in liquid minimal media. Upon exposure to Congo red, increased MpkA phosphorylation was observed in the wild-type strain, while it remained unchanged in the Δ*sas3* mutant, suggesting that loss of Sas3 impacts the MpkA phosphorylation. Taken together, these results suggested that Sas3 is important for the maintenance of cell wall structure and CWI pathway in *A. fumigatus*.

### The function of Sas3 is affected by mutations in the combination of three conserved glycine and glutamate residues

**Fig 5:**
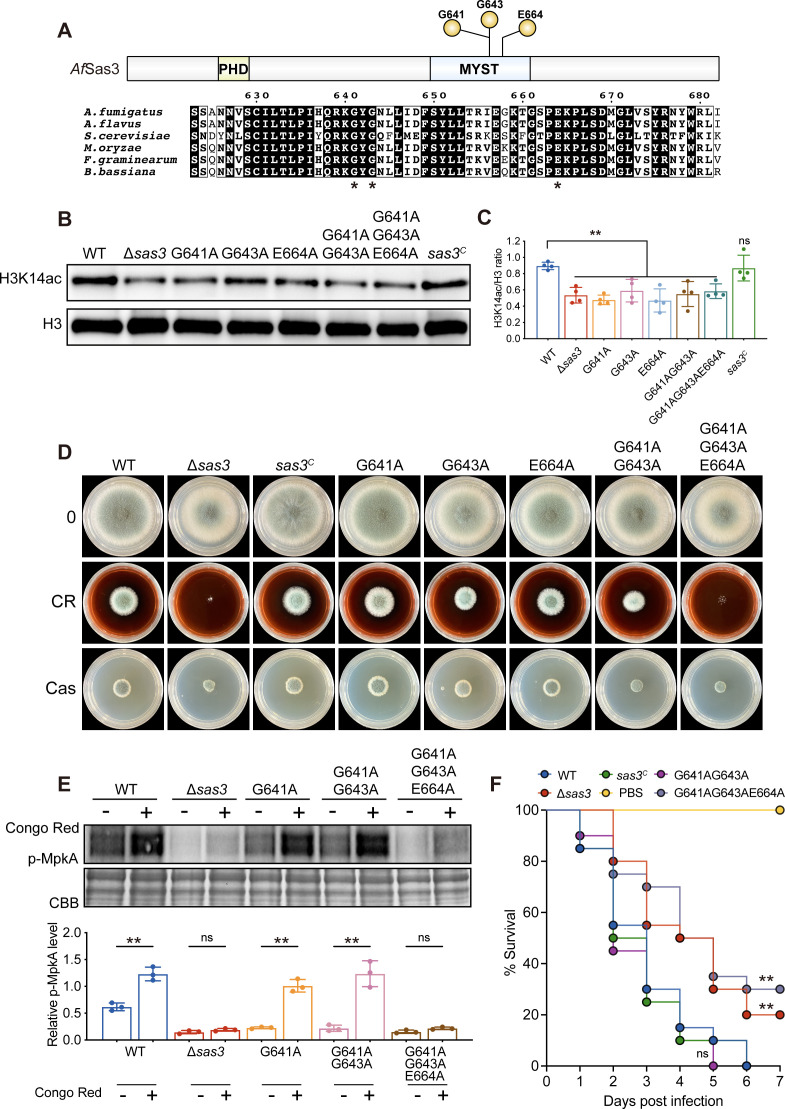
The conserved glycine and glutamate residues are critical for the functions of Sas3. (**A**) Schematic diagram of the *A. fumigatus* Sas3 protein. The green box represents the PHD domain, and the blue box indicates the MYST domain. Sequence alignment of Sas3 orthologs performed with Clustal Omega. The conserved glycine and glutamate residues are marked with asterisks. (**B**) Western blotting for acetylated H3K14 in the wild-type, Δ*sas3*, site-directed mutants, and complementation strains. H3 served as a loading control. (**C**) Quantified analysis of Western blot signal for H3K14ac relative to H3 in the indicated strains. Data from three independent experiments are presented as mean ± SD. Statistical analysis was performed using one-way analysis of variance with multiple comparisons tests. (**D**) Colony morphology of the indicated strains grown on solid YG medium in the presence of 200-µg/mL Congo red and 0.5-µg/mL caspofungin at 37°C for 48 h. (**E**) Western blot analysis of p-MpkA in the wild-type and Δ*sas3* mutants in the presence and absence of CR. CBB staining was used as a loading control (upper panel) and quantification of signal intensity ratio for p-MpkA to actin. Data are shown as mean ± SD from three independent experiments. Statistical analysis was performed using two-tailed, unpaired *t*-tests (lower panel). (**F**) Survival curves for *G. mellonella* larvae infected with the indicated strains. Each experiment was replicated three times independently. Statistical analysis was using the log-rank test. **P < 0.01. CBB, Coomassie brilliant blue; H3, histone H3; ns, not significant; p-MpkA, phosphorylation levels of MpkA.

To further analyze the molecular characteristics of Sas3 in *A. fumigatus*, we performed a sequence alignment of Sas3 with its orthologs in other species (25, 30, 31), and this analysis revealed that two glycine residues (G641 and G643) and one glutamate residue (E664) were evolutionarily conserved (Fig. 5A). We then introduced exogenously mutated *sas3*^G641A^, *sas3*^G643A^, and *sas3*^E664A^ genes to the Δ*sas3* background to construct the single, double, and triple point mutations and to characterize the function of these residues in *A. fumigatus*. The qRT-PCR analysis revealed that the expression of the mutated genes was similar to that of the wild-type *sas3* gene, suggesting that the transcription levels of the mutated genes remained unaffected (Fig. S2). Western blot analysis revealed a significant decrease in acetylation of histone H3K14 in all *sas3* point mutations compared to the wild-type and complementation strains (Fig. 5B and C), suggesting that the conserved glycine and glutamate are required for the histone acetyltransferase activity of Sas3 in *A. fumigatus*. Interestingly, the phenotypic analysis revealed that only triple point mutation resembled that of the Δ*sas3* in the presence and absence of cell wall-perturbing agents, while single point mutations G641A, G643A, and E664A and double point mutation G641A*/*G643A showed a similar phenotype to the wild-type strain (Fig. 5D). In line with the colony morphology, only triple point mutation G641/G643/E664 showed a pattern of MpkA phosphorylation level similar to that of the Δ*sas3* mutant with or without cell wall stress (Fig. 5E). In addition, the G641/G643/E664 triple mutant exhibited a significant reduction in virulence, while the G641/G643 double mutant displayed mortality rates similar to those of the wild-type strain (Fig. 5F). Collectively, these results suggested that the mutations in the combination of conserved glycine and glutamate residues affect the functions of Sas3.

To gain insight into the mechanism underlying Sas3-mediated cell wall integrity, we next performed an affinity pull-down assay coupled with liquid chromatography-tandem mass spectrometry (LC-MS/MS) to identify the potential Sas3-interacting proteins (Table S4). As shown in Table 1, we identified several previously reported Sas3-interacting proteins: Nto1, Yng1, Taf14, and Eaf6. These proteins are components of the NuA3 complex (17, 32–34). This result validated the reliability of the experiment. Importantly, Rho guanyl nucleotide exchange factor Rom2 (35) and protein kinase C PkcA (36) were identified as potential Sas3-interacting proteins. Rom2 and PkcA have been shown to play an essential role in the cell wall integrity of *A. fumigatus*. These results implied that Sas3 may regulate the cell wall integrity through interaction with Rom2 and PkcA.

**TABLE 1:** Putative Sas3-interacting proteins from FLAG pull-down assay

Gene ID	Protein name	Description	Molecular weight (kDa)
AFUB_067970	Sas3	Histone acetyltransferase	114.9
AFUB_090830	Nto1	NuA3 complex component	133.2
AFUB_037250	Yng1	NuA3 complex component	77.1
AFUB_087590	Adh3	Alcohol dehydrogenase	37.5
AFUB_027190	Rrp8	rRNA processing protein	57.6
AFUB_054310	CpcC	Putative protein kinase for translation initiation factor eIF2	179.7
AFUB_059780	Taf14	NuA3 complex component	25.9
AFUB_040920	Eaf6	NuA3 complex component	26.3
AFUB_056090	Rom2	Rho guanyl nucleotide exchange factor	135.6
AFUB_059540	PkcA	Protein kinase C	123.1
AFUB_035330	NsdD	Sexual development transcription factor	53.0
AFUB_018960	ChsA	Chitin synthase A	104.1

## DISCUSSION

Histone acetylation is crucial for the regulation of transcriptional reprogramming across a wide range of biological processes (7, 8, 37). However, the functions of specific modified histone acetyltransferase that are involved in this modification remain largely unknown in filamentous fungi. In this work, we investigated the role of the MYST family HAT Sas3 in the development, virulence, and cell wall integrity of human pathogen *A. fumigatus*.

Previous studies have shown that MYST-family proteins are highly conserved and exert crucial regulatory functions in development, conidiation, and secondary metabolic processes across various fungal species, such as plant pathogenic fungi *Zymoseptoria tritici* (23), *M. oryzae* (20), *F. graminearum* (22), *Botrytis cinerea* (38), and opportunistic pathogen *A. flavus* (25). In agreement with prior findings, we found that deletion of *sas3* leads to a significant reduction in hyphal growth and conidiation when compared to the wild-type strain (Fig. 1B and C). These results underscore the indispensable role of Sas3 for vegetative growth and conidiation in *A. fumigatus*. Importantly, the *A. fumigatus* Δ*sas3* mutant was found to be less virulent compared to the wild-type strain in the *G. mellonella* model, resulting in an increased host survival rate (Fig. 1D). We cannot exclude the possibility that the reduced virulence phenotype may be attributed in part to the impairments in hyphal growth observed in *A. fumigatus* Δ*sas3* mutant *in vitro*. It is worth noting that although there is a good correlation between the virulence of several fungal pathogens in *G. mellonella* and in mammalian models (39, 40), further research needs to be conducted with animal models to validate these findings.

As the catalytic subunit of acetyltransferase complex NuA3, Sas3 has been demonstrated to be necessary for the acetylation of histone H3 on K9, K14, K18, and K23 (17, 18, 25). Likewise, we showed that loss of Sas3 resulted in decreased acetylation of histone H3K9 and H3K14 by Western blot assay using specific anti-acetylated lysine antibodies (Fig. 2D). Moreover, subcellular localization showed that Sas3 localizes specifically to the nucleus in *A. fumigatus*, providing further evidence of its role as a HAT (Fig. 2B). Collectively, these results indicated that Sas3 functions as bona fide histone acetyltransferase in *A. fumigatus* and plays an important role in the acetylation of H3K9 and H3K14. Lysine 9 and 14 of histone H3 have been demonstrated to be acetylated by various HATs such as Gcn5, Rtt109, and Esa1 (14, 30, 41). This explains why deletion of *sas3* failed to completely abolish the acetylation of H3K9 and H3K14. The major function of histone acetylation is the regulation of gene transcription. A global transcriptomic profile revealed that Sas3 affects several important biological pathways, such as carbohydrate metabolism, amino acid biosynthesis, fatty acid biosynthesis, and glycolysis, highlighting the regulatory role of Sas3 in *A. fumigatus* (Fig. 3A and B). Therefore, it is reasonable to believe that the defective phenotypes observed in the Δ*sas3* mutant are mainly caused by the misregulation of the transcriptional network. Notably, among the differentially expressed genes in the Δ*sas3* mutant, we identified several genes that are essential for the cell wall integrity (CWI) pathway. These genes include *mpkC*, *ssk1*, and *steC*, all of which are significantly downregulated (Fig. 3C). Consistent with the downregulated expression of these CWI genes, the Δ*sas3* mutant strain exhibited cell wall defects, demonstrated by increased sensitivity to cell wall-perturbing agents compared to the parental strain, an increase in cell wall thickness and aberrant phosphorylation levels of MpkA, a MAP kinase that is required for cell wall organization (Fig. 4). These results indicated the significance of Sas3 in the maintenance of cell wall architecture and the CWI pathway in *A. fumigatus*.

The pleiotropic phenotypes observed in the Δ*sas3* mutant prompt the question of whether alterations in histone acetylation contribute to defects in development and cell wall stress response. To address this question, we performed multiple sequence alignments to identify the conserved residues that are presumably involved in the catalytic activity of Sas3 (Fig. 5A). We constructed the single, double, and triple mutant strains for the conserved sites G641, G643, and E664 separately. *In vivo* HAT assay showed that all point mutations in *sas3* led to a decrease in the acetylation of histone H3K14, demonstrating the essential role of these conserved residues in the histone acetyltransferase activity of Sas3 (Fig. 5B and C). Surprisingly, only G641A/G643A/E664A triple mutations led to the growth defects and exhibited increased sensitivity to cell wall stress, altered phosphorylation levels of MpkA, and attenuated virulence, which resembled that of the Δ*sas3* mutant (Fig. 5D, E and F). In contrast, the single mutants G641A, G643A, and E664A, as well as the double mutant G641A/G643A showed no discernible difference compared to the wild-type strain under the same condition (Fig. 5D, E and F), indicating that only the combination of three conserved glycine and glutamate residues could give rise to the defective phenotypes of *A. fumigatus*. In *A. flavus*, a mutation at a single residue E243, which corresponds to residue E664 in *A. fumigatus* Sas3, is sufficient to impair colony growth, conidiation, sclerotia formation, aflatoxin production, and pathogenicity, resembling the effects observed in the null mutant (25). These inconsistent results raise the possibility that the defective phenotypes may be attributed to structural or other functions that are independent of the HAT activity of Sas3. Indeed, our FLAG pull-down assay showed that Sas3 may physically interact with Rom2 and PkcA, the key components of the CWI pathway (Table 1). Future work will further verify whether Sas3 affects the functions of Rom2 and PkcA through non-histone acetylation.

In conclusion, we demonstrate that MYST-family histone acetyltransferase Sas3 is indispensable for fungal development, virulence, and cell wall integrity in *A. fumigatus*. Our findings will expand our knowledge of the biological functions of histone acetyltransferase in human fungal pathogens and shed light on the development of a novel strategy for fungal disease control.

## MATERIALS AND METHODS

### Strains, media, and culture conditions

The *A. fumigatus* strains used in this study are listed in Table S1. The *A. fumigatus* strains were cultivated on two types of media: MM and rich medium (YG). MM was composed of 10-g/L glucose, 50-mL/L 20× salts, and 1-mL/L 1,000× trace elements, supplemented with 2% agar. YG consisted of 20-g/L glucose, 1-mL/L 1,000× trace elements, 5-g/L yeast extract, and 2% agar (42). All liquid media were prepared based on the corresponding solid media without agar. Fresh *A. fumigatus* conidia on solid YG plates were collected with sterile water. Strains were incubated at 37°C unless otherwise stated.

### Construction of strains

The primers used in this paper are listed in Table S2. To obtain the *sas3*-null mutant strain, the complete open reading frame of the target gene was replaced with the resistance gene hygromycin B phosphotransferase (*hph*) through homologous recombination. Generally, the homologous arms of the target gene were amplified using Sas3-P1/P3 and Sas3-P4/P6 primers, which were derived from *A. fumigatus* genomic DNA with a length of approximately 1,500 bp. Additionally, a fragment of the *hph* gene, which acts as a resistance screening marker, was amplified by using the pAN7-1 plasmid along with hph-F and hph-R primers. Finally, the three fragments were combined by fusion PCR using Sas3-P2/P5 primers and transformed into the *A. fumigatus* KU80 protoplast.

To construct *sas3* complementation strain, the *sas3* gene fragment was amplified using the sas3-phle complementation-F/R primer with *A. fumigatus* genomic DNA as template, and the phleomycin gene fragment was amplified using the phle-F/R primers with pTATA plasmid as template. Primers sas3-phle complementation-F and phle-R were used to fuse the *sas3* gene with the phleomycin gene by fusion PCR. Subsequently, the fusion fragment was transformed into the protoplast of the recipient Δ*sas3* mutant.

To construct the Sas3-GFP tagged strain, the left and right homologous arms of the *sas3* gene were amplified from the genomic DNA at approximately 1,500 bp using primers Sas3-GFP-P1/P3 and Sas3-GFP-P4/P6, respectively. It is worth noting that the termination codon of the *sas3* gene is located outside of the left homology arm. The fusion fragment of GFP and pyrG was amplified from the plasmid pFNO3 using primers GFP + pyrG F/R, which were called gfp + pyrG fragments. The three fragments were fused using primers Sas3-GFP-P2/P5 and then transferred into protoplasts of the recipient A1160. The same procedure used to generate the Sas3-GFP strain was used to generate the Sas3-FLAG strain.

To generate *sas3* point mutant strains, we employed specially designed upstream and downstream primers with distinct point mutation sites. The plasmid containing the *sas3* complementation fragment obtained from the experiments on the construction of the *sas3* complementation strain served as the template for acquiring the respective linear point mutant gene fragments. Then, the plasmid template was digested with DpnI for 2 h at 37°C. The resulting linear fragment was recycled using recombinase. Subsequently, the mutated site on the circular plasmid was introduced into *Escherichia coli* Trans1-T1 (TransGen Biotech) and then transformed into *sas3* null mutant. Transformation of *A. fumigatus* was conducted according to previous protocols (43). The selection markers hygromycin B and phleomycin were used at concentrations of 200 and 150 µg/mL, respectively.

### Phenotypic analysis of strains

To analyze the morphology of the colonies and the growth of the mycelium, equal amounts of 2 µL of the conidia suspension (1 × 10^7^ conidia/mL) were spotted on solid MM and YG plates, respectively, and incubated at 37°C for 48 h. Colony diameter was measured after 48 h incubation. To determine conidia production, all conidia were harvested from solid plates incubated for 48 h at 37°C and counted using a hemacytometer. To determine biomass, 1 mL of conidia suspension with a concentration of 1 × 10^8^ conidia/mL was aspirated and inoculated into 100 mL of liquid MM medium, incubated at 37°C, 220 rpm for 24 h. The mycelium was collected with gauze squeezed dry and then dried in an incubator at 65°C; the dry weight of the mycelium was weighed as biomass. To test the sensitivity of the strains to the cell wall stress reagents, 2 µL of 1 × 10^7^ conidia/mL conidia suspension was aspirated and plated equally on YG solid plates containing the cell wall stress reagents CFW (200 µg/mL), CR (300 µg/mL), and Cas (0.5 µg/mL), respectively. The colonies were then cultured in an incubator at 37°C for 48 h to observe the growth phenotypes of the colonies.

### *G. mellonella* virulence assay

The *G. mellonella* virulence assay was performed using a method described previously (44). Fresh conidia of the corresponding strains were harvested and adjusted to 1 × 10^8^ conidia/mL. Ten microliters of conidia suspension was then injected into the *G. mellonella* larvae through left prolegs. The control group was injected with a sterile phosphate-buffered saline (PBS) solution. *G. mellonella* larvae were then incubated in darkness at 37°C for 7 days, and the number of larval deaths was recorded every 24 h to calculate the survival rate. The significance of virulence among the various strains was analyzed. Each experiment was replicated three times independently, and each replication contained 20 larvae per strain.

### Fluorescence microscopy

To visualize the localization of Sas3-GFP fusion protein, 1 × 10^6^ conidia were cultured on glass coverslips in liquid MM at 37°C for 9 h. After discarding the medium, the coverslips were rinsed thoroughly with PBS three times, followed by the application of 4% (vol/vol) paraformaldehyde for 30 min at room temperature. Subsequently, the paraformaldehyde was removed, and another round of rinsing with PBS was carried out. Then, the coverslips were immersed in PBS containing a 1-µg/mL Hoechst dye 33258 solution (Sangon Biotech, E607301) and kept in darkness for 30 min. To visualize the chitin content in the cell wall, samples were stained with 5-mg/mL CFW (Maokang Biotechnology, MS4040) in PBS for 1 min. Fluorescence microscopy was conducted after the incubation using a Zeiss Axio imager A1 microscope (Carl Zeiss, Germany) to capture the images.

### Quantitative real-time PCR and RNA sequencing

Total RNA for qRT-PCR was extracted using the Total RNA Extraction Kit UNIQ-10 (Sango Biotech, B511361) according to the instructions. RNA (500 ng) was used as a template, and the cDNA was synthesized via reverse transcription using HiScript II Q RT SuperMix for qPCR (Vazyme, R222-01). cDNA obtained by reverse transcription was used as a template for qRT-PCR, and the housekeeping gene tubulin (*tubA*) was used as an internal reference gene in the ABI one-step fast thermocycler (Applied Biosystems) with Hieff qPCR SYBR Green Master Mix (Yeasen, 11203ES08). The relative expression of genes was determined using the ΔΔCт method with normalization to *tubA* expression (45). The RNA sequencing of both the wild-type and Δ*sas3* mutants was conducted using the Illumina platform (Personalbio, Shanghai, China).

### Cell wall thickness analysis

1 mL of conidia suspension of fresh wide-type and Δ*sas3* strains at a concentration of 10^8^ conidia/mL was inoculated into 100 mL of liquid MM medium and incubated at 37°C, 220 rpm for 24 h. A portion of the mycelium was taken and soaked in glutaraldehyde and sent to the company (Wuhan Service Biotechnology) for scanning transmission electron microscopy.

### Protein extraction and Western blotting

The relevant strains were inoculated into 100 mL of liquid MM medium and incubated at 37°C, 220 rpm for 24 h. Mycelium was collected with gauze, squeezed dry, and then quickly placed in a bucket of liquid nitrogen. Subsequently, the mycelium was ground in liquid nitrogen with a mortar and pestle to obtain the powder. The specific steps for protein extraction were described previously (46). Protein samples were added to SDS-PAGE gels for separation, and then the proteins were transferred to a polyvinylidene difluoride (PVDF) membrane (Millipore). The PVDF membrane was then incubated at low speed under room temperature for 1.5 h in 5% skimmed milk [prepared with phosphate-buffered saline with Tween (PBST) solution], washed with PBST solution, and rotated overnight at 4°C in the primary antibody solution. The primary antibodies used in this study are as follows: anti-H3 (Sigma, H9289), anti-H3K56ac (Sigma, 07–677), anti-H3K9ac (PTM-Bio, PTM-156), anti-H3K14ac (Abclonal, A7254), rabbit anti-GFP-Tag mAb (Abclonal, AE078), DDDDK-tag Rabbit mAb (FLAG) (Abclonal, AE092), β-actin rabbit mAb (Abclonal, AC026), and phospho-ERK1-T202/Y204 + ERK2 T185/Y187 rabbit mAb (Abclonal, AP0974). After the primary antibody incubation, the membrane was washed three times with PBST solution for 10 min each time, and then the PVDF membrane was immersed in the corresponding secondary antibody solution and incubated at low speed for 1 h at room temperature. The secondary antibodies and dilution ratios used in this paper are horseradish peroxidase (HRP) goat anti-mouse IgG (H + L) (Abclonal, AS003) and HRP goat anti-rabbit IgG (H + L) (Abclonal, AS014). At the end of the incubation, the membrane was rinsed once more with PBST solution, and then the Enhanced ECL luminescence detection kit (Vazyme, E411) was used for exposure. The images were captured using a Tanon 4200 chemiluminescence imaging system (Tanon). The band intensity was calculated by ImageJ software.

### Sas3 affinity pull-down assay

Mycelium of the Sas3-FLAG strain was collected and ground into powder, and 1 mL of lysis buffer [containing 10-mM Tris-HCl, pH 7.5, 150-mM NaCl, 0.5-mM EDTA, 1-mM dithiothreitol (DTT), 0.01% Triton X-100, 1-mM phenylmethylsulfonyl fluoride (PMSF), and protease inhibitor mixture] (Beyotime, P1020) was used to extract total protein. Anti-FLAG M2 agarose beads (Sigma-Aldrich, A2220) were gently mixed with 5 mg of total protein sample and purified according to the manufacturer’s instructions. Liquid chromatography-tandem mass spectrometry was utilized to detect and analyze the samples.

### Statistical analysis

Statistical analyses were performed using GraphPad Prism version 9.0 (GraphPad software, USA). Two-tailed unpaired Student’s *t*-test was used to compare differences between two groups. One-way analysis of variance was used for comparison between multiple groups. The log-rank (Mantel-Cox) test was used to compare survival curves. A *P* value of <0.01 was considered statistically significant. The data are shown as mean ± SD from at least three independent experiments.

## Data Availability

The RNA-seq data were deposited in the National Center for Biotechnology Information Sequence Read Archive database under accession number PRJNA1030550. The complete data set is presented in the supplemental material.

## SUPPLEMENTAL MATERIAL

Fig. S1 aem.01885-23-s0001.tif Construction of sas3 mutant.

Fig. S2 aem.01885-23-s0002.tif mRNA expression of sas3 mutant.

Fig. S1 and S2 legends, Tables S1 and S2, Table S3 and S4 legends aem.01885-23-s0003.docx Supplemental tables and legends.

Table S3 aem.01885-23-s0004.xlsx Differentially expressed genes between the wild-type and Δsas3 strains.

Table S4 aem.01885-23-s0005.xlsx The putative Sas3-interacting proteins identified by the FLAG pull-down assay.

ASM does not own the copyrights to Supplemental Material that may be linked to, or accessed through, an article. The authors have granted ASM a non-exclusive, world-wide license to publish the Supplemental Material files. Please contact the corresponding author directly for reuse.

## References

[1] Millán-Zambrano G, Burton A, Bannister AJ, Schneider R. 2022. Histone post-translational modifications - cause and consequence of genome function. Nat Rev Genet 23:563–580. doi:10.1038/s41576-022-00468-7 35338361

[2] Li X, Li XD. 2021. Integrative chemical biology approaches to deciphering the histone code: a problem-driven journey. Acc Chem Res 54:3734–3747. doi:10.1021/acs.accounts.1c00463 34553920

[3] Musselman CA, Lalonde M-E, Côté J, Kutateladze TG. 2012. Perceiving the epigenetic landscape through histone readers. Nat Struct Mol Biol 19:1218–1227. doi:10.1038/nsmb.2436 23211769 PMC3645987

[4] Bannister AJ, Kouzarides T. 2011. Regulation of chromatin by histone modifications. Cell Res 21:381–395. doi:10.1038/cr.2011.22 21321607 PMC3193420

[5] Marmorstein R, Zhou MM. 2014. Writers and readers of histone acetylation: structure, mechanism, and inhibition. Cold Spring Harb Perspect Biol 6:a018762. doi:10.1101/cshperspect.a018762 24984779 PMC4067988

[6] Lai Y, Wang L, Zheng W, Wang S. 2022. Regulatory roles of histone modifications in filamentous fungal pathogens. J Fungi (Basel) 8:565. doi:10.3390/jof8060565 35736048 PMC9224773

[7] Jeon J, Kwon S, Lee YH. 2014. Histone acetylation in fungal pathogens of plants. Plant Pathol J 30:1–9. doi:10.5423/PPJ.RW.01.2014.0003 25288980 PMC4174833

[8] Shen Y, Wei W, Zhou DX. 2015. Histone acetylation enzymes coordinate metabolism and gene expression. Trends Plant Sci 20:614–621. doi:10.1016/j.tplants.2015.07.005 26440431

[9] van de Veerdonk FL, Gresnigt MS, Romani L, Netea MG, Latgé J-P. 2017. Aspergillus fumigatus morphology and dynamic host interactions. Nat Rev Microbiol 15:661–674. doi:10.1038/nrmicro.2017.90 28919635

[10] Arastehfar A, Carvalho A, Houbraken J, Lombardi L, Garcia-Rubio R, Jenks JD, Rivero-Menendez O, Aljohani R, Jacobsen ID, Berman J, Osherov N, Hedayati MT, Ilkit M, Armstrong-James D, Gabaldón T, Meletiadis J, Kostrzewa M, Pan W, Lass-Flörl C, Perlin DS, Hoenigl M. 2021. Aspergillus fumigatus and aspergillosis: from basics to clinics. Stud Mycol 100:100115. doi:10.1016/j.simyco.2021.100115 34035866 PMC8131930

[11] Latgé J-P, Chamilos G. 2019. Aspergillus fumigatus and aspergillosis in 2019. Clin Microbiol Rev 33:e00140-18. doi:10.1128/CMR.00140-18 31722890 PMC6860006

[12] Gu Q, Wang Y, Zhao X, Yuan B, Zhang M, Tan Z, Zhang X, Chen Y, Wu H, Luo Y, Keller NP, Gao X, Ma Z. 2022. Inhibition of histone acetyltransferase GCN5 by a transcription factor FgPacC controls fungal adaption to host-derived iron stress. Nucleic Acids Res 50:6190–6210. doi:10.1093/nar/gkac498 35687128 PMC9226496

[13] Chen M, Liu Y, Liu Z, Su L, Yan L, Huang Y, Huang Y, Zhang W, Xu X, Zheng F. 2023. Histone acetyltransferase Gcn5-mediated histone H3 acetylation facilitates cryptococcal morphogenesis and sexual reproduction. mSphere 8. doi:10.1128/msphere.00299-23 PMC10732044 37850793

[14] Zhang Y, Fan J, Ye J, Lu L. 2021. The fungal‐specific histone acetyltransferase Rtt109 regulates development, DNA damage response, and virulence in Aspergillus fumigatus. Mol Microbiol 115:1191–1206. doi:10.1111/mmi.14665 33300219

[15] Sun R, Wen M, Wu L, Lan H, Yuan J, Wang S. 2021. The fungi-specific histone acetyltransferase Rtt109 mediates morphogenesis, aflatoxin synthesis and pathogenicity in Aspergillus flavus by acetylating H3K9. IMA Fungus 12:9. doi:10.1186/s43008-021-00060-4 33823938 PMC8025522

[16] Wurtele H, Tsao S, Lépine G, Mullick A, Tremblay J, Drogaris P, Lee E-H, Thibault P, Verreault A, Raymond M. 2010. Modulation of histone H3 lysine 56 acetylation as an antifungal therapeutic strategy. Nat Med 16:774–780. doi:10.1038/nm.2175 20601951 PMC4108442

[17] John S, Howe L, Tafrov ST, Grant PA, Sternglanz R, Workman JL. 2000. The something about silencing protein, Sas3, is the catalytic subunit of NuA3, a yTAF(II)30-containing HAT complex that interacts with the Spt16 subunit of the yeast CP (Cdc68/Pob3)-FACT complex. Genes Dev 14:1196–1208. 10817755 PMC316621

[18] Church M, Smith KC, Alhussain MM, Pennings S, Fleming AB. 2017. Sas3 and Ada2(Gcn5)-dependent histone H3 acetylation is required for transcription elongation at the de-repressed FLO1 gene. Nucleic Acids Res 45:4413–4430. doi:10.1093/nar/gkx028 28115623 PMC5416777

[19] Takechi S, Nakayama T. 1999. Sas3 is a histone acetyltransferase and requires a zinc finger motif. Biochem Biophys Res Commun 266:405–410. doi:10.1006/bbrc.1999.1836 10600516

[20] Dubey A, Lee J, Kwon S, Lee YH, Jeon J. 2019. A MYST family histone acetyltransferase, MoSAS3, is required for development and pathogenicity in the rice blast fungus. Mol Plant Pathol 20:1491–1505. doi:10.1111/mpp.12856 31364260 PMC6804344

[21] Wang JJ, Cai Q, Qiu L, Ying SH, Feng MG. 2018. The histone acetyltransferase Mst2 sustains the biological control potential of a fungal insect pathogen through transcriptional regulation. Appl Microbiol Biotechnol 102:1343–1355. doi:10.1007/s00253-017-8703-9 29275430

[22] Kong X, van Diepeningen AD, van der Lee TAJ, Waalwijk C, Xu J, Xu J, Zhang H, Chen W, Feng J. 2018. The Fusarium graminearum histone acetyltransferases are important for morphogenesis, DON biosynthesis, and pathogenicity. Front Microbiol 9:654. doi:10.3389/fmicb.2018.00654 29755419 PMC5932188

[23] Suarez-Fernandez M, Álvarez-Aragón R, Pastor-Mediavilla A, Maestre-Guillén A, Del Olmo I, De Francesco A, Meile L, Sánchez-Vallet A. 2023. Sas3-mediated histone acetylation regulates effector gene activation in a fungal plant pathogen. mBio 14:e0138623. doi:10.1128/mbio.01386-23 37642412 PMC10653901

[24] Fan A, Mi W, Liu Z, Zeng G, Zhang P, Hu Y, Fang W, Yin WB. 2017. Deletion of a histone acetyltransferase leads to the pleiotropic activation of natural products in Metarhizium robertsii. Org Lett 19:1686–1689. doi:10.1021/acs.orglett.7b00476 28301168

[25] Chen X, Wu L, Lan H, Sun R, Wen M, Ruan D, Zhang M, Wang S. 2022. Histone acetyltransferases MystA and MystB contribute to morphogenesis and aflatoxin biosynthesis by regulating acetylation in fungus Aspergillus flavus. Environ Microbiol 24:1340–1361. doi:10.1111/1462-2920.15856 34863014

[26] Ram AFJ, Klis FM. 2006. Identification of fungal cell wall mutants using susceptibility assays based on Calcofluor white and Congo red. Nat Protoc 1:2253–2256. doi:10.1038/nprot.2006.397 17406464

[27] Kahn JN, Hsu MJ, Racine F, Giacobbe R, Motyl M. 2006. Caspofungin susceptibility in Aspergillus and non-Aspergillus molds: inhibition of glucan synthase and reduction of beta-D-1,3 glucan levels in culture. Antimicrob Agents Chemother 50:2214–2216. doi:10.1128/AAC.01610-05 16723587 PMC1479154

[28] Sanz AB, García R, Rodríguez-Peña JM, Arroyo J. 2017. The CWI pathway: regulation of the transcriptional adaptive response to cell wall stress in yeast. J Fungi (Basel) 4:1. doi:10.3390/jof4010001 29371494 PMC5872304

[29] Valiante V, Jain R, Heinekamp T, Brakhage AA. 2009. The MpkA MAP kinase module regulates cell wall integrity signaling and pyomelanin formation in Aspergillus fumigatus. Fungal Genet Biol 46:909–918. doi:10.1016/j.fgb.2009.08.005 19715768

[30] Decker PV, Yu DY, Iizuka M, Qiu Q, Smith MM. 2008. Catalytic-site mutations in the MYST family histone acetyltransferase Esa1. Genetics 178:1209–1220. doi:10.1534/genetics.107.080135 18245364 PMC2278108

[31] Yan Y, Harper S, Speicher DW, Marmorstein R. 2002. The catalytic mechanism of the ESA1 histone acetyltransferase involves a self-acetylated intermediate. Nat Struct Biol 9:862–869. doi:10.1038/nsb849 12368900

[32] Taverna SD, Ilin S, Rogers RS, Tanny JC, Lavender H, Li H, Baker L, Boyle J, Blair LP, Chait BT, Patel DJ, Aitchison JD, Tackett AJ, Allis CD. 2006. Yng1 PHD finger binding to H3 trimethylated at K4 promotes NuA3 HAT activity at K14 of H3 and transcription at a subset of targeted ORFs. Mol Cell 24:785–796. doi:10.1016/j.molcel.2006.10.026 17157260 PMC4690528

[33] Howe L, Kusch T, Muster N, Chaterji R, Yates JR III, Workman JL. 2002. Yng1p modulates the activity of Sas3p as a component of the yeast NuA3 Hhistone acetyltransferase complex. Mol Cell Biol 22:5047–5053. doi:10.1128/MCB.22.14.5047-5053.2002 12077334 PMC139787

[34] Eberharter A, John S, Grant PA, Utley RT, Workman JL. 1998. Identification and analysis of yeast nucleosomal histone acetyltransferase complexes. Methods 15:315–321. doi:10.1006/meth.1998.0635 9740719

[35] Samantaray S, Neubauer M, Helmschrott C, Wagener J. 2013. Role of the guanine nucleotide exchange factor Rom2 in cell wall integrity maintenance of Aspergillus fumigatus. Eukaryot Cell 12:288–298. doi:10.1128/EC.00246-12 23264643 PMC3571289

[36] Jackson-Hayes L, Atiq Z, Betton B, Freyaldenhoven WT, Myers L, Olsen E, Hill TW. 2019. Aspergillus nidulans protein kinase C forms a complex with the formin SepA that is involved in apical growth and septation. Fungal Genet Biol 122:21–30. doi:10.1016/j.fgb.2018.10.002 30391723

[37] Audia JE, Campbell RM. 2016. Histone modifications and cancer. Cold Spring Harb Perspect Biol 8:a019521. doi:10.1101/cshperspect.a019521 27037415 PMC4817802

[38] Wang G, Song L, Bai T, Liang W. 2020. BcSas2-mediated histone H4K16 acetylation is critical for virulence and oxidative stress response of Botrytis cinerea Mol Plant Microbe Interact 33:1242–1251. doi:10.1094/MPMI-06-20-0149-R 32689887

[39] Durieux MF, Melloul E, Jemel S, Roisin L, Darde ML, Guillot J, Dannaoui E, Botterel F. 2021. Galleria mellonella as a screening tool to study virulence factors of Aspergillus fumigatus. Virulence 12:818-834. 33682618 10.1080/21505594.2021.1893945 PMC7946008

[40] Slater JL, Gregson L, Denning DW, Warn PA. 2011. Pathogenicity of Aspergillus fumigatus mutants assessed in Galleria mellonella matches that in mice. Med Mycol 49 Suppl 1:S107–13. doi:10.3109/13693786.2010.523852 20950221

[41] Miao J, Wang C, Lucky AB, Liang X, Min H, Adapa SR, Jiang R, Kim K, Cui L. 2021. A unique GCN5 histone acetyltransferase complex controls erythrocyte invasion and virulence in the malaria parasite Plasmodium falciparum. PLoS Pathog 17:e1009351. doi:10.1371/journal.ppat.1009351 34403450 PMC8396726

[42] Todd RB, Davis MA, Hynes MJ. 2007. Genetic manipulation of Aspergillus nidulans: heterokaryons and diploids for dominance, complementation and haploidization analyses. Nat Protoc 2:822–830. doi:10.1038/nprot.2007.113 17446882

[43] Szewczyk E, Nayak T, Oakley CE, Edgerton H, Xiong Y, Taheri-Talesh N, Osmani SA, Oakley BR. 2006. Fusion PCR and gene targeting in Aspergillus nidulans. Nat Protoc 1:3111–3120. doi:10.1038/nprot.2006.405 17406574

[44] Zhang Y, Wang Y, Fan J, Zhu G, Lu L. 2022. Aspergillus fumigatus Elongator complex subunit 3 affects hyphal growth, adhesion and virulence through wobble uridine tRNA modification. PLoS Pathog. 18:e1010976. doi:10.1371/journal.ppat.1010976 36374932 PMC9704764

[45] Pfaffl MW. 2001. A new mathematical model for relative quantification in real-time RT-PCR. Nucleic Acids Res 29:e45. doi:10.1093/nar/29.9.e45 11328886 PMC55695

[46] Long N, Orasch T, Zhang S, Gao L, Xu X, Hortschansky P, Ye J, Zhang F, Xu K, Gsaller F, Straßburger M, Binder U, Heinekamp T, Brakhage AA, Haas H, Lu L. 2018. The Zn2Cys6-type transcription factor LeuB cross-links regulation of leucine biosynthesis and iron acquisition in Aspergillus fumigatus. PLoS Genet 14:e1007762. doi:10.1371/journal.pgen.1007762 30365497 PMC6221358

