# Supplementary figures and images for: Histone acetyltransferase Sas3 contributes to fungal development, cell wall integrity, and virulence in Aspergillus fumigatus
Source: Appl Environ Microbiol. 2024 Mar 7;90(4):e01885-23. doi: 10.1128/aem.01885-23 (PMC11022558; doi:10.1128/aem.01885-23)

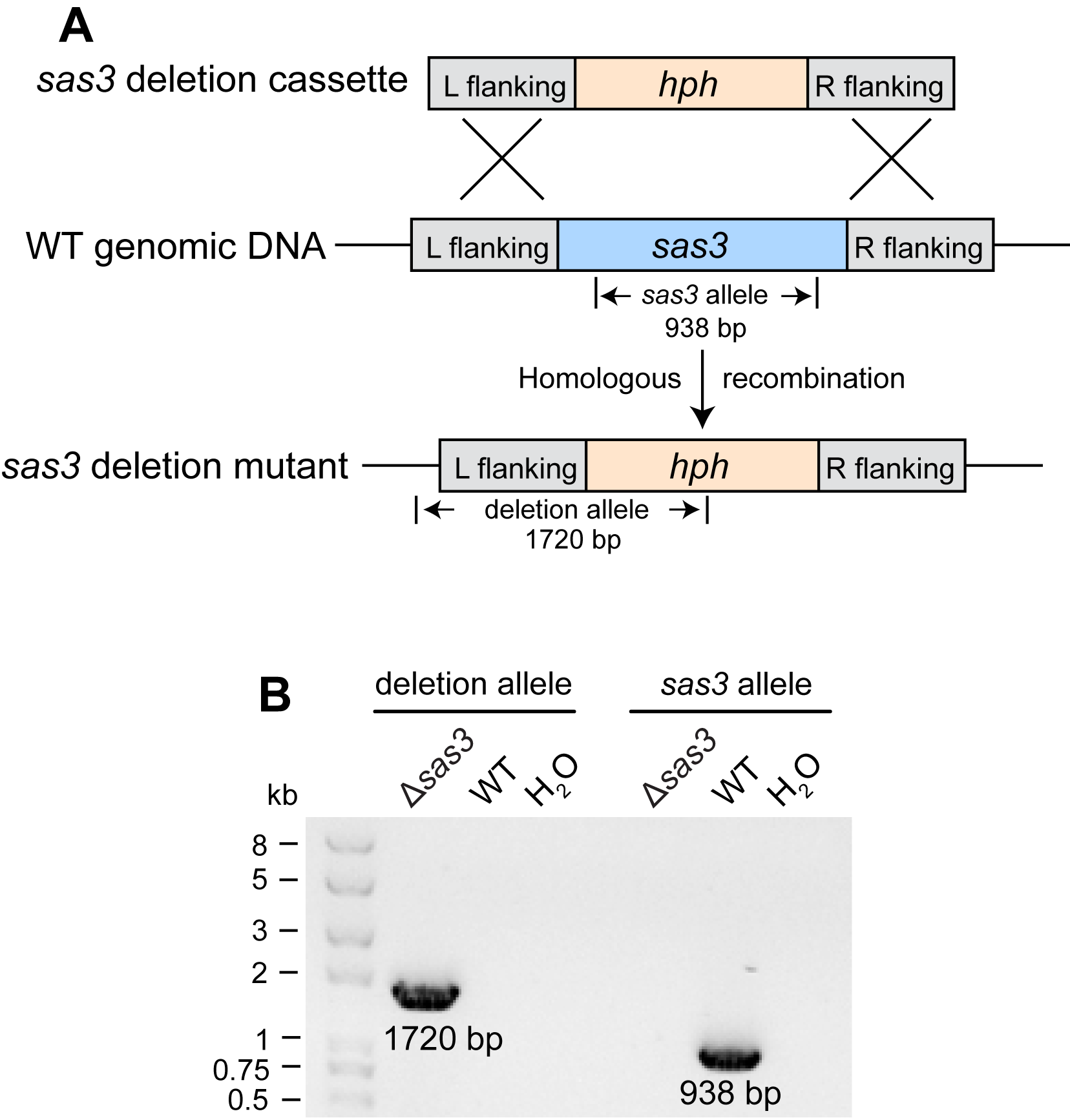

Supplement: Fig. S1 — Construction of sas3 mutant. [file aem.01885-23-s0001.tif]

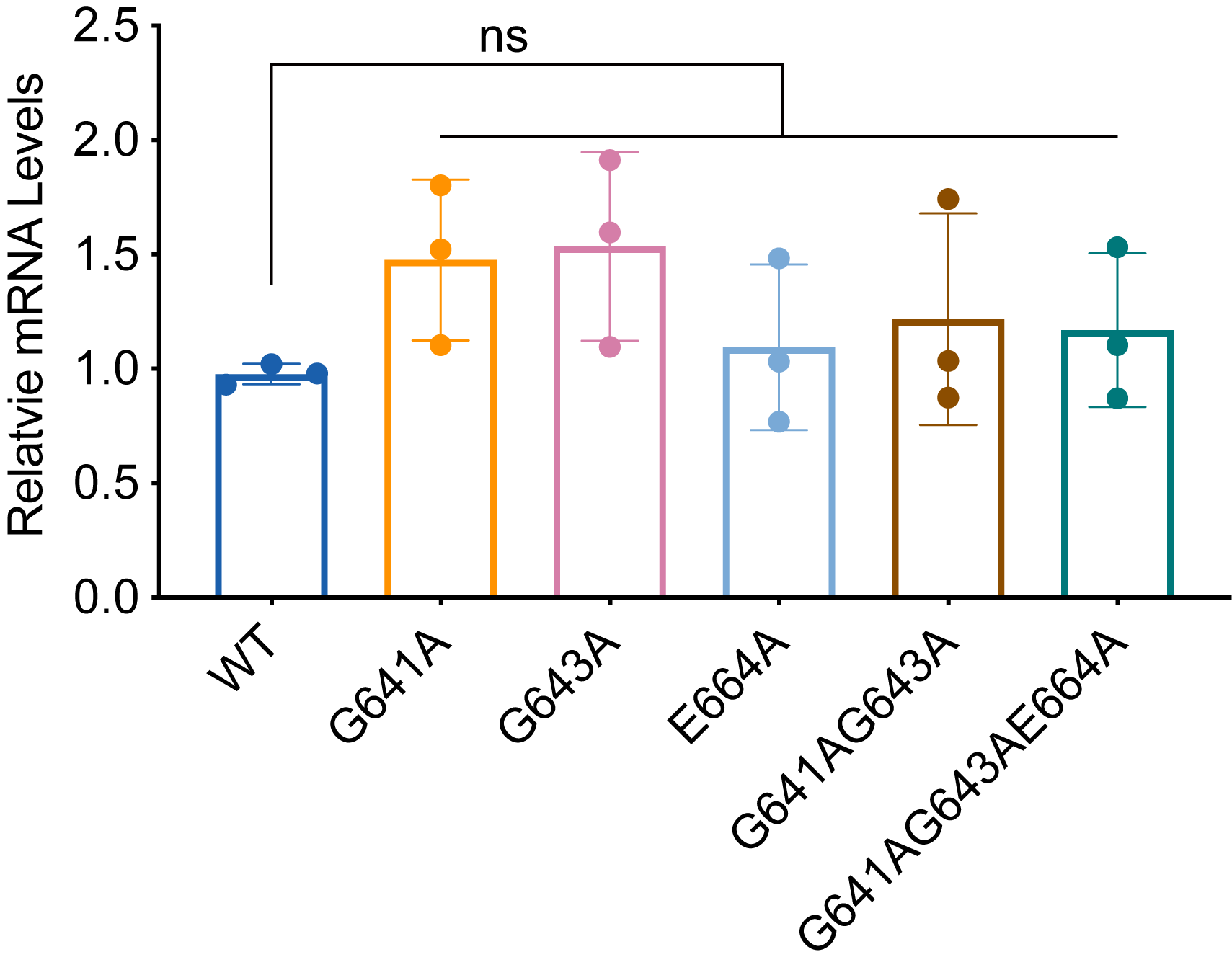

Supplement: Fig. S2 — mRNA expression of sas3 mutant. [file aem.01885-23-s0002.tif]
