# Supplementary material for: Histone acetyltransferase Sas3 contributes to fungal development, cell wall integrity, and virulence in Aspergillus fumigatus
Source: Appl Environ Microbiol. 2024 Mar 7;90(4):e01885-23. doi: 10.1128/aem.01885-23 (PMC11022558; doi:10.1128/aem.01885-23)
Supplement: Fig. S1 and S2 legends, Tables S1 and S2, Table S3 and S4 legends — Supplemental tables and legends. [file aem.01885-23-s0003.docx]

**Supplemental materials**

**Figure Legends**

**Figure S1. Construction of the *sas3* null mutant.**

(A) Diagram illustrating the targeted gene homologous replacement for the *sas3* gene.

(B) Diagnostic PCR confirmed the homologous integration at the original *sas3* locus in the Δ*sas3* strain.

**Figure S2. mRNA expression analysis of *sas3*^G641A^, *sas3*^G643A^ and *sas3*^E664A^ in the Δ*****sas3* mutant.**

Expression analysis of *sas3*^G641A^, *sas3*^G643A^ and *sas3*^E664A^ in the Δ*sas3* strains grown in liquid MM at 37°C for 48 h by qRT-PCR. The mRNA levels were normalized to an mRNA level of the reference gene *tubA*. Data are shown as mean ± SD from three independent experiments.

**Table S1. Strains used in this study**

| **Strain** | **Genotype** | **Source** |
| --- | --- | --- |
| A1160 | Δ*ku80*, *pyrG* | FGSC |
| KU80 | Δ*ku80* | (1) |
| Δ*sas3* | Δ*ku80,* Δ*sas3::hph* | This study |
| *sas3^C^* | Δ*ku80,* Δ*sas3::hph,* *sas3::* *phle* | This study |
| Sas3-GFP | Δ*ku80,* *pyrG,* *sas3::GFP::pyrG* | This study |
| Sas3-FLAG | Δ*ku80,* *pyrG,* *sas3::FLAG::pyr4* | This study |
| G641A | Δ*ku80,* Δ*sas3::hph,* *sas3G641A::phle* | This study |
| G643A | Δ*ku80,* Δ*sas3::hph,* *sas3^G643A^::phle* | This study |
| E664A | Δ*ku80,* Δ*sas3::hph,* *sas3^E664A^::phle* | This study |
| G641A/G643A | Δ*ku80,* Δ*sas3::hph,* *sas3^G641A/G643A^::* *phle* | This study |
| G641A/G643A/E664A | Δ*ku80,* Δ*sas3::hph,* *sas3^G641/AG643A/E664A^::phle* | This study |

**Table S2. Primers used in this study.**

| Primer | Sequence (5’- 3’) |
| --- | --- |
| Sas3-P1 | CGTTTCGCTTGCCCAGTAAT |
| Sas3-P2 | GGTTGCTGACATCGAATCCC |
| Sas3-P3 | CGGCGGATTTTAGGCTCAAGGGTTTCGCGATGTTTGAACG |
| Sas3-P4 | GTTGCCTAGTGAATGCTCCGATAGGAAACGGGAGGGCAAA |
| Sas3-P5 | GGCACAGGGGACATCTTAGA |
| Sas3-P6 | CGAAATGAGGGGAGCACAAG |
| hph-F | CTTGAGCCTAAAATCCGCCG |
| hph-R | CGGAGCATTCACTAGGCAAC |
| Sas3-self-F | AGATGCGGACACCTCAAGAA |
| Sas3-self-R | AGCCTTTCTCCTCCCAGTTC |
| HPH-R | CTGCCGGTGATTCGATGAAG |
| sas3-phle-F | AAGAGATCGAACCAGGCGTC |
| sas3-phle-R | TAATCAATTGCCCGTCTGTCAACTGCACTGCCTTGACTCTA |
| phle-F | TGACAGACGGGCAATTGATTA |
| phle-R | AGGAGGGCGTGAATGTAAGC |
| Phle-R | CCATGACTTCCATCGTATGCC |
| GFP+pyrG-F | GGAGCTGGTGCAGGCGCTGG |
| GFP+pyrG-R | CTGTCTGAGAGGAGGCACTGATG |
| Sas3-GFP-P1 | GGGTGCTCTACATCTGCGA |
| Sas3-GFP-P2 | AAGGCTACGGGAATTTGCTT |
| Sas3-GFP-P3 | CCAGCGCCTGCACCAGCTCCTGTCTGCATGACTGCGTCG |
| Sas3-GFP-P4 | CATCAGTGCCTCCTCTCAGACAGTGACAATGCTTGACAACCAGA |
| Sas3-GFP-P5 | ATACTCTGCACTGCCCATGT |
| Sas3-GFP-P6 | ATGATGAACAAGTTCAAATCCCG |
| GFP+pyrG-R | TAGGGACCGAGACCTGTATC |
| FLAG+pyr4-F | CTCGAGGGATCCCCGGGAATG |
| FLAG+pyr4-R | GCTTTCGGGAACTGGCTACTTAT |
| Sas3-FLAG-P3 | CATTCCCGGGGATCCCTCGAGTGTCTGCATGACTGCGTCG |
| Sas3-FLAG-P4 | ATAAGTAGCCAGTTCCCGAAAGCTGACAATGCTTGACAACCAGA |
| pyr4-R | GGTCCAAGTGGAAGTAGGTAGTGAC |
| Sas3-G641A-F | AAGAAAAGCCTACGGGAATTTGCTTATCGACT |
| Sas3-G641A-R | TCCCGTAGGCTTTTCTTTGATGGATGGGCAGT |
| Sas3-G643A-F | AGGCTACGCGAATTTGCTTATCGACTTCTCATACC |
| Sas3-G643A-R | GCAAATTCGCGTAGCCTTTTCTTTGATGGATG |
| Sas3-E664A-F | TCGCCAGCGAAACCTCTCTCCGACATGGGCCT |
| Sas3-E664A-R | AGAGGTTTCGCTGGCGAGCCAGTCTTACCTTC |
| RT-brlA-F | GGGCCATACGGAGTCGATTG |
| RT-brlA-R | GGCGAGTGCGTCTTGAAGGT |
| RT-abaA-F | GACTGGCAGCCCGAGTGTATT |
| RT-abaA-R | GTCATCACGACCACTCATCCC |
| RT-wetA-F | CCCATCAGTCACCGCAACC |
| RT-wetA-R | GGGAGGAGTGGACGGTGAT |
| RT-mpkC-F | GAGGTCTTGGGTACGAAGTTC |
| RT-mpkC -R | ACTGGTTGCTGGGTTATCTG |
| RT-ssk1-F | AGTCCCGTTATCATTGTCGC |
| RT-ssk1-R | CCCATTCTGTCACCTTTTGTTC |
| RT-steC-F | TTATGAGAAAAGACCCTCCGC |
| RT-steC-R | AGGGAAGATGCAGAATTCGG |
| RT-sho1-F | TCTCCGTCCTTTCATAAACCG |
| RT-sho1-R | GATACATGCGATCAACCACG |
| RT-tcsB-F | CCGATTGAGGGACATGGTATT |
| RT-tcsB-R | AATTGCGTCGAGAGTCCTG |
| RT-gel2-F | ACGAGCAGAGTGTCAAGAAC |
| RT-gel2-R | GTAGTTGAGGGTGTCCATGAG |
| RT-gel3-F | CCTGCTATCTGGATGTGCTATC |
| RT-gel3-R | CGTAGTTCGCTTCAGACTGG |
| RT-nagA-F | CTATCTTGACTGTGGCTCTGG |
| RT-nagA-R | GTTGAAGTTTGGTGTGCTGG |
| RT-097510-F | ACAAGCAACCTACTCGACAC |
| RT-097510-R | ATTTCGTCTTCCCATTCCCC |
| RT-029980-F | AGCAACGACATGACTGGTAC |
| RT-029980-R | GTTCCCTTGACTACACATCCG |
| RT-tub-F | TTCCGTCCCGACAACTTCGT |
| RT-tub-R | TCACAGCCTTCAGCCTCACG |

**Table S3. Differentially expressed genes between the wild-type and Δ*sas3* strains.**

**Table S4. The putative Sas3-interacting proteins identified by the FLAG pull-down assay.**

**References**

1. da Silva Ferreira ME, Kress MR, Savoldi M, Goldman MH, Hartl A, Heinekamp T, Brakhage AA, Goldman GH. 2006. The akuB(KU80) mutant deficient for nonhomologous end joining is a powerful tool for analyzing pathogenicity in *Aspergillus fumigatus*. Eukaryot Cell 5:207-11.
